# Induction of allopurinol resistance in *Leishmania infantum* isolated from dogs

**DOI:** 10.1371/journal.pntd.0005910

**Published:** 2017-09-11

**Authors:** Daniel Yasur-Landau, Charles L. Jaffe, Adi Doron-Faigenboim, Lior David, Gad Baneth

**Affiliations:** 1 Koret School of Veterinary Medicine, The Hebrew University, Rehovot, Israel; 2 Department of Microbiology and Molecular Genetics, IMRIC, The Hebrew University–Hadassah Medical Center, Jerusalem, Israel; 3 Agricultural Research Organization, The Volcani Center, Institute of Plant Science, Bet Dagan, Israel; 4 Department of Animal Sciences, The Hebrew University, Rehovot, Israel; Institute of Tropical Medicine, BELGIUM

## Abstract

Resistance to allopurinol in zoonotic canine leishmaniasis has been recently shown to be associated with disease relapse in naturally-infected dogs. However, information regarding the formation of resistance and its dynamics is lacking. This study describes the successful *in-vitro* induction of allopurinol resistance in *Leishmania infantum* cultured under increasing drug pressure. Allopurinol susceptibility and growth rate of induced parasites were monitored over 23 weeks and parasite clones were tested at selected time points and compared to their parental lines, both as promastigotes and as amastigotes. Allopurinol resistance was formed in strains from two parasite stocks producing a 20-fold rise in IC_50_ along three distinct growth phases. In addition, characteristic differential clustering of single nucleotide polymorphisms (SNP) was found in drug sensitive and resistant parasite clones. Results confirm that genetic polymorphism, as well as clonal heterogeneity, contribute to *in-vitro* resistance to allopurinol, which is likely to occur in natural infection.

## Author summary

Visceral leishmaniasis caused by the parasite *Leishmania infantum* is a neglected tropical disease transmitted from animal hosts to humans by sand fly bites. This potentially fatal disease affects thousands of people annually and threatens millions who live in disease risk areas. Domestic dogs are considered as the main reservoir of this parasite which can also cause a severe chronic canine disease. Allopurinol is the main drug used for long term treatment of this disease but it often does not eliminate infection in dogs. We have recently demonstrated that allopurinol resistant parasites can be isolated from naturally infected dogs that have developed clinical recurrence of disease during allopurinol treatment. In this study we aimed to see if resistance can be induced in susceptible parasite strains isolated from sick dogs by growing them in increasing drug concentrations under laboratory conditions. The changes in allopurinol susceptibility were measured and the impact of drug on parasite growth was monitored over 23 weeks. Induction of resistance was successful producing parasites 20-folds less susceptible to the drug. The pattern of change in drug susceptibility suggests that a genetic change is responsible for the increased resistance which is likely to mimic the formation of resistance in dogs.

## Introduction

Visceral leishmaniasis caused by *Leishmania infantum* is a life threatening disease, affecting humans in Europe, Asia, North Africa and Latin America, as well as domestic dogs which are the main reservoir for this infection [1, 2]. We recently reported the detection of disease relapse in infected dogs associated with allopurinol resistant parasite strains [3]. Allopurinol is the main drug used for long-term control of the canine disease, and since resistant parasites may enhance transmission to humans and other dogs [4], this finding is alarming. In this study, we aimed to improve our understanding of the formation of resistance to allopurinol by following an *in-vitro* model of resistance induction in susceptible isolates under increasing drug pressure and examining the susceptibility to allopurinol of several clones from the same time point, in both promastigote and amastigote stages.

## Methods

### Parasite cultures

Two allopurinol susceptible *L*. *infantum* isolates, obtained prior to drug treatment from dogs at time of first diagnosis of clinical disease, were used in the study; MCAN/IL/2011/NT4 and MCAN/IL/2011/NT5. Both dogs were males; presented weight loss, skin lesions, enlarged lymph nodes, mild anemia and elevated serum globulin levels. Dog NT4 was also azotemic. Isolation, culture procedure and IC_50_ testing were done as previously described [3]. Briefly, allopurinol susceptibility was determined using a promastigote viability test, following 72 h incubation in increasing drug concentrations. Each test was repeated twice.

### Induction of allopurinol resistance

Resistance was induced in cultures designated NT4.L and NT5.L in a stepwise manner; beginning with the original isolates and every 2–6 days thereafter, 5*10^6^ promastigotes were transferred into 5 mL of complete M-199 medium containing increasing allopurinol concentrations, starting at 100 μg/mL with 50 μg/mL increments. Thus, each step was defined as the period between two successive subcultures. Average growth rate for each step was calculated as the increase in parasite concentration during the step divided by its length in days (average step growth rate–ASGR). Once parasites were able to grow at 900 μg/mL allopurinol, they were maintained in it for at least two additional months. Allopurinol IC_50_ of cultures was tested every 7–14 days and culture samples were cryopreserved. Controls of each isolate cultured without allopurinol, designated NT4 and NT5, were maintained and tested in parallel. All culture medium components manufacturers and lot numbers were kept constant for the duration of the experiments.

### Drug susceptibility testing in promastigote and amastigote clones

Drug induced cultures from selected time points were thawed and single clones were isolated using the hanging drop method, adapted from Evans and Smith [5]. Briefly, 0.5 μL samples were taken of each culture adjusted to contain 2*10^3^ parasites per mL. Samples were inspected microscopically, and those containing individual promastigotes were subcultured in 200 μL of culture medium until a stable clonal culture was established. IC_50_ was established for each revived frozen culture, as well as for 5–10 of its clones. Allopurinol susceptibility was also studied in intracellular amastigotes developed for each induced strain and its clones at one time point, as previously described [3]. Briefly, DH-82 cells were infected with promastigotes from thawed samples of the drug induced strains and 5 respective clonal cultures. Infected cells were treated with either 0 or 300 μg/mL allopurinol for 72h, followed by counting of intracellular parasites per 100 DH-82 cells on Giemsa stained preparations, and calculation of the percent inhibition caused by the drug. Drug-free control cultures were also thawed and tested at the specific time points.

### Whole genome sequencing and SNP's analysis of clonal cultures

Six clonal strains derived from cultures NT4.L and NT5.L as described above, presenting low (n = 2) and high (n = 4) allopurinol IC_50_ values were chosen for whole genome sequencing (WGS). These included clone 1 of NT4.L on day 28 (NT4.L.s, see S1 Table) and clones 3 and 4 from day 104 (NT4.L.r1 and NT4.L.r2, respectively); clone 2 of NT5.L from day 28 (NT5.L.s) and clones 1 and 5 of day 86 (NT5.L.r1 and NT5.L.r2, respectively).

DNA for WGS was extracted from 2*10^8^ mid log-phase promastigotes of each of the six strains described above. Following centrifugation at 1500 rpm for 10 minutes, supernatant was discarded and promastigotes were suspended in 250μL phosphate buffered saline. DNA was then extracted using the Illustra blood genomicPrep Mini Spin KIT (GE Healthcare, UK) according to manufacturer’s instructions and included RNAse treatment (RNAse A, Sigma-Aldrich, St. Louis, MO). Quantity and quality of DNA was tested using the NanoDrop 2000 (Thermo Scientific, Wilmington, DE), followed by visualization in 1% agarose gel with ethidium bromide. Fragmentation was done using the Covaris shearing (Covaris S2, Covaris, Woburn, MA) set to the size target at 400bp for library preparation. Libraries were made using the TruSeq DNA kit (Genomic DNA Sample Prep Kit, FC-102-1004, Illumina, San Diego, CA).

Sequencing was done using 100 bases paired ends reads, on an Ilumina HiSeq2000 platform, with the TruSeq SBS Kit (TruSeq SBS v3, FC-401-3001, Illumina, San Diego, CA) and TruSeq PE Cluster Kit (TruSeq PE Cluster Kit v3, PE-401-3001, Illumina, San Diego, CA), at the DNA LandMarks Laboratory (St.-Jean-sur-Richelieu, Canada).

Raw reads were subjected to a cleaning procedure using the FASTX Toolkit (http://hannonlab.cshl.edu/fastx_toolkit/index.html, version 0.0.13.2). Trimming read end nucleotides with quality scores under 30 was done using the fastq_quality_trimmer, and removal of read pairs was done if reads in the read pair had less than 70% base pairs with quality score under or equal to 23, using the fastq_quality_filter.

The *L*. *infantum* JPCM5 genome with chromosomes 1–36 was used as a reference genome (European nucleotide archive, BioProject PRJNA12658, FR796433—FR796468). Cleaned paired-end reads, obtained after processing and cleaning of the 6 samples were mapped to the reference genome using the Bowtie2 program version 2.0.0 with default parameters [6]. SNP analysis was done using the Picard (http://broadinstitute.github.io/picard/) and GATK UnifiedGenotyper (version 2.5–2) [7] programs. The SNP’s calling was done in reference to the *L*. *infantum* JPCM5 genome. The degree of similarity between SNP of the six induced resistant strains was studied by a maximum likelihood analysis with bootstrapping (N = 100), using the PhyML 3.0 software [8].

### Statistical analysis

Comparing IC_50_ values between induced and control cultures for matching time points was done using the t-test. Tukey HSD and Wilcoxon tests were used to compare IC_50_ values within each drug-cultured isolate at different time points. The Tukey HSD test was used to compare IC_50_ and percent inhibition values within strains and respective clones on each time point. Correlations between promastigote IC_50_ values and amastigote percent inhibition values for respective clones were described for NT4.L and NT5.L using a linear, logarithmic or polynomial trend lines.

### Ethics statement

The animal care protocol used in this study was approved by the Hebrew University’s Institutional Animal Care and Use Committee (IACUC); approval no. MD-08-11476-2, following the USA NIH guidelines.

## Results

### Induction of allopurinol resistance

**Fig 1:**
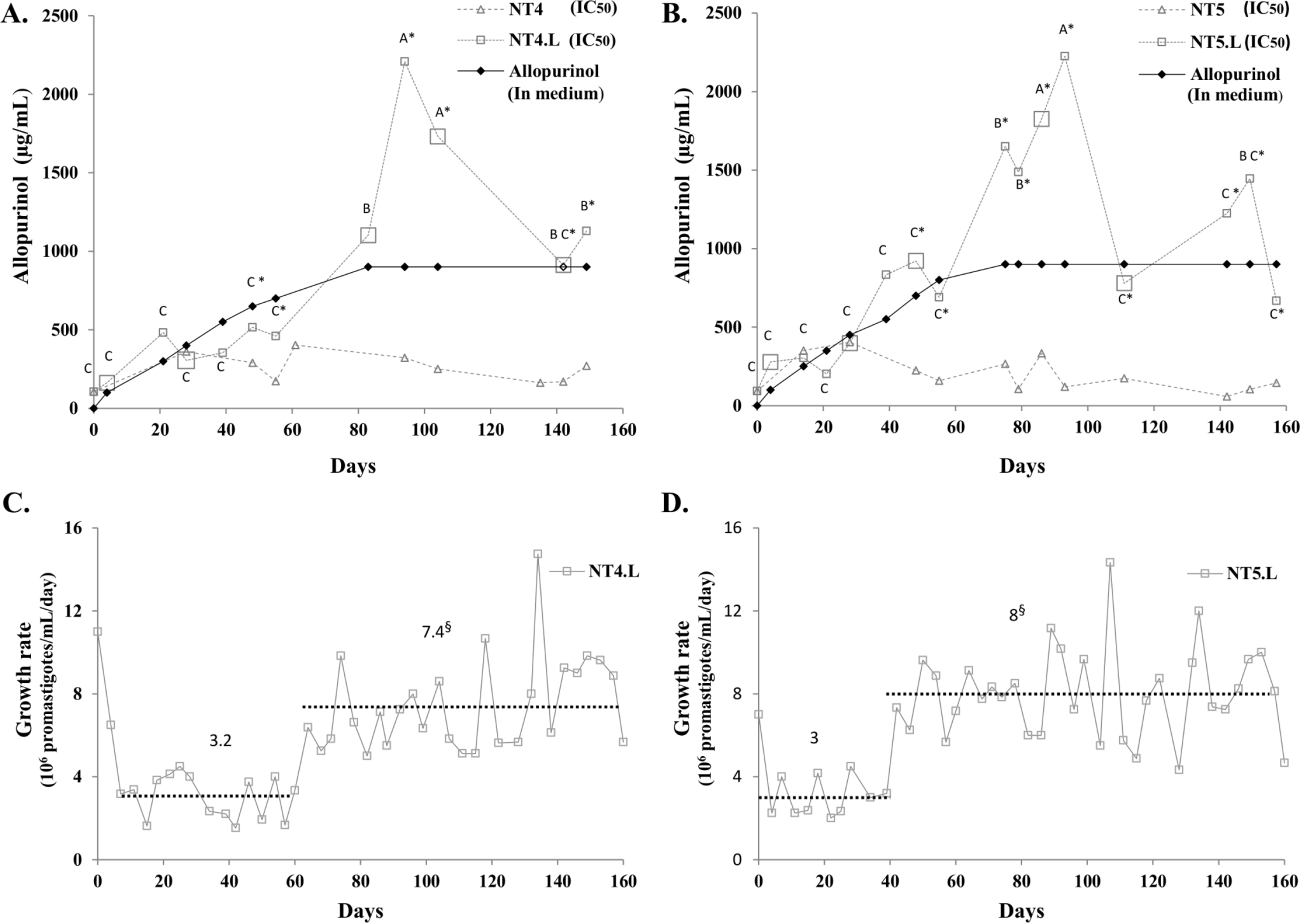
Changes in allopurinol susceptibility and growth rates in *Leishmania infantum* cultures NT4.L and NT5.L under drug pressure. (A, B) IC_50_ of promastigotes cultured under drug pressure (NT4.L, NT5.L) as compared to controls (NT4, NT5) cultured in medium alone over time. Only IC_50_ values with R square >0.9 are presented. The allopurinol concentrations in medium are also shown. Distinct letters refer to significant differences in IC_50_ values between time points within each induced culture, NT4.L (A) or NT5.L (B). Asterisk (*) refers to differences in IC_50_ values between the induced and respective control culture (for e.g. NT4.L and NT4) for the same time point. Large squares indicate points of cloning. (C, D) Average step growth rate (ASGR) values for cultures under drug pressure, showing two distinct phases. Values expressed in 10^6^ promastigotes/mL/day. Dotted lines and labels represent average ASGR values for respective phase. § Difference in ASGR averages between phases.

A starting allopurinol concentration of 100 μg/mL was chosen because the IC_50_ values of the parent isolates were 105 and 93 μg/mL for NT4 and NT5, respectively [3]. During the experiment, drug concentration in the culture medium quadrupled by day 22 and maximal drug level tested was 900 μg/mL allopurinol from days 60–71 and on, about 10 folds higher than the initial IC_50_ values of NT4.L and NT5.L, and close to the average IC_50_ value found for resistant clinical isolates previously [3] (Fig 1A and 1B).

Allopurinol susceptibility kinetics showed three distinguishable phases in both isolates. In the initial phase (up to day 28 in NT5.L and day 60 in NT4.L), IC_50_ values had not changed significantly compared to the initial value, and were comparable to those measured for the controls at most points. In the second phase, a significant 4 folds increase in IC_50_ was seen, only in drug exposed cultures of both isolates (t-test, *P* <0.05). Peak IC_50_ values recorded were 2225 μg/mL at day 93 for NT5.L and 2209 μg/mL at day 94 for NT4.L. In the third phase, a decline of over 2 folds in IC_50_ compared to peak values was measured for both drug-cultured isolates (Tukey HSD and Wilcoxon tests, *P* <0.05). Control cultures NT4 and NT5, not exposed to drug pressure, fluctuated in IC_50_ over time, however peak values did not exceed 405 μg/mL.

Using ASGR values, two distinct phases were detected in both induced cultures, marked by a sharp change in ASGR. In the first phase, growth rate was decreased compared to the original isolate, and slower growth lasted approximately to day 39 for NT5.L (allopurinol concentration 550 μg/mL) or day 60 for NT4.L (allopurinol concentration 700 μg/mL). In the second phase, growth rate significantly increased by 2 folds or more (Fig 1C and 1D, t-test, *P* <0.001). Difference in frequencies of steps length between the two growth phases was tested, and no significant difference was found between the two phases (χ^2^ test, *P* = 0.3945), confirming that promastigotes growth rates were indeed increased in phase two for both cultures.

### Drug susceptibility testing in promastigote and amastigote clones

**Fig 2:**
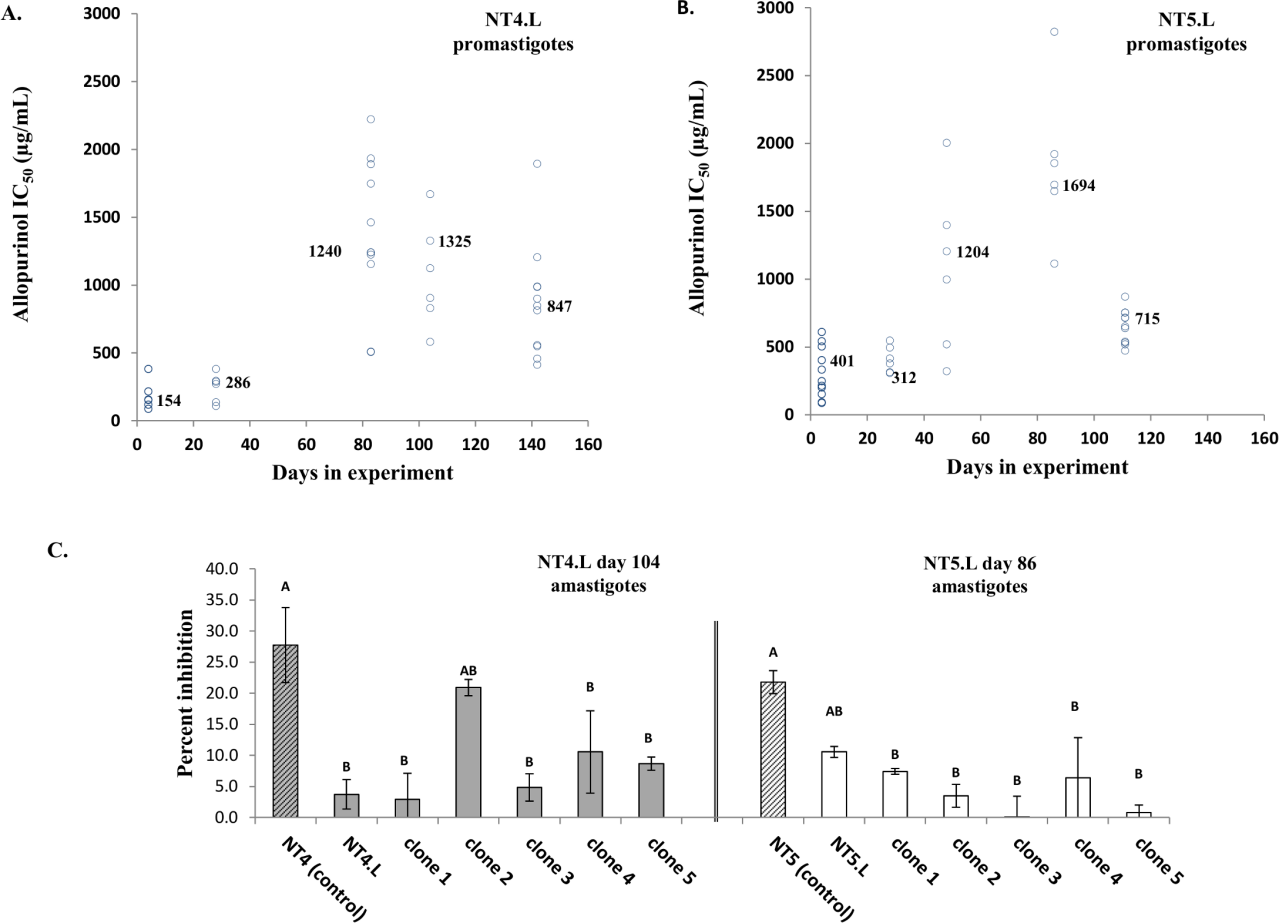
Allopurinol susceptibility of *L*. *infantum* parental strains and clones at different time points during induction of resistance. (A, B) Results for promastigotes, numbers indicate IC_50_ values of the post-thaw parent cultures at the respective time points. See also S1 Table. (C) Allopurinol percent inhibition results for intracellular amastigotes. An induced resistant culture, 5 of its respective clones and its drug-free control culture were tested for each parental line (NT4.L and NT5.L) at one time point (day 104 and 86, respectively). Values indicated by distinct letters differ significantly (Tukey HSD test, p<0.05).

Frozen drug-cultured isolate samples from different time points were revived, clones were isolated and their promastigote IC_50_ compared with that of the respective parent sample (Fig 2A and 2B).

IC_50_ values of clones presented a 2–4 fold variation at all time points, with significant differences detected between clones (Tukey HSD test, *P* <0.05, S1 Table). Interestingly, values of parent cultures were within the 95% confidence intervals created by values of their respective clones. As found for promastigotes, testing of intracellular amastigotes also demonstrated in most cases a significant difference between susceptible control strains and resistant induced strains and clones (Fig 2C, S2 Table). Inter-clonal variation demonstrated in amastigotes was smaller than seen in promastigotes. However, this can be in part due to limitation of the assay that prevents using drug concentrations of over 300μg/mL allopurinol. R square values for correlation between promastigote IC_50_ values and amastigote percent inhibition values ranged between 0.86–0.97 for NT4.L and 0.7–0.79 for NT5.L, for linear and polynomial model, respectively.

**Fig 3:**
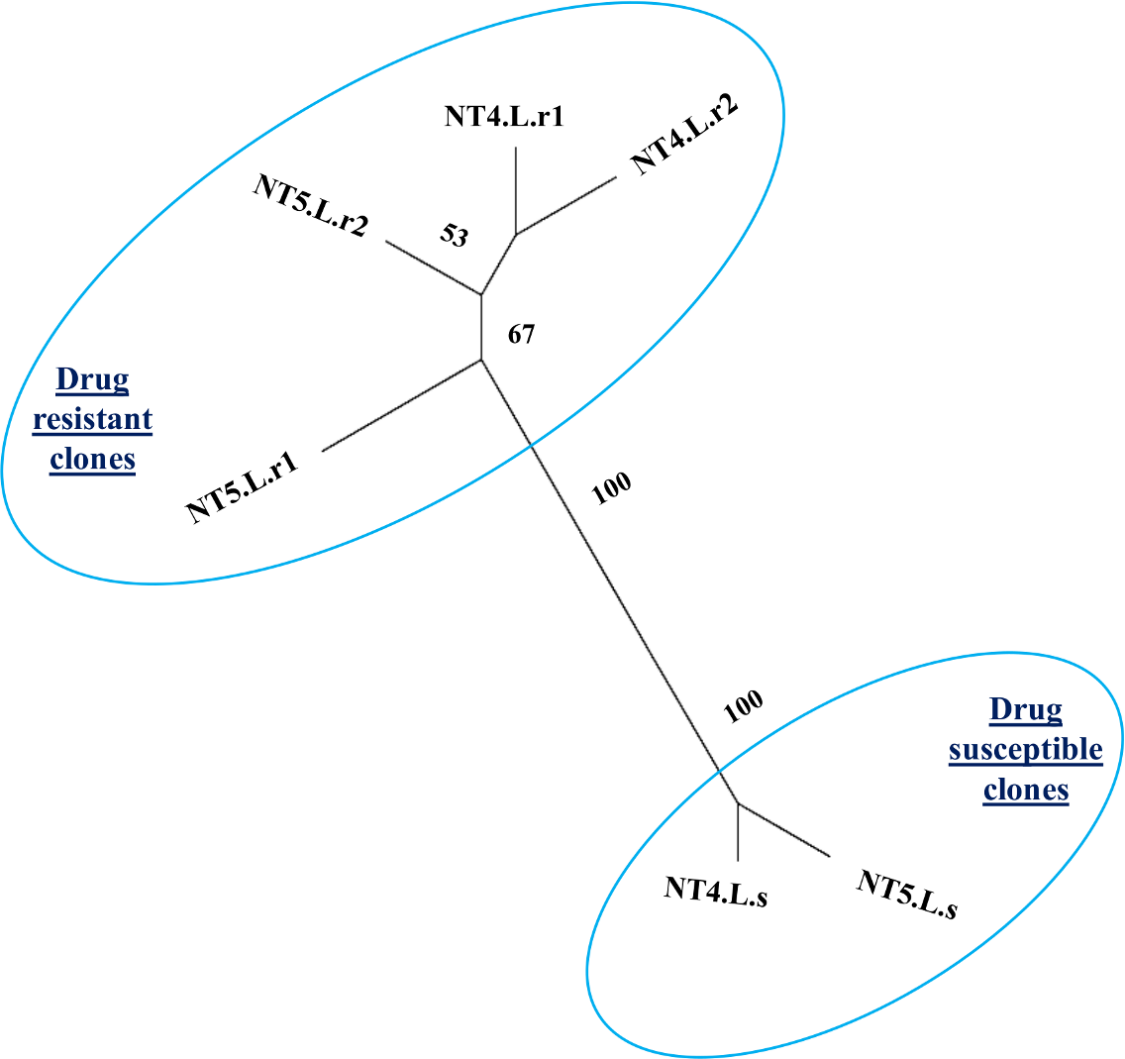
Maximum likelihood analysis of six induced-resistance clonal strains for all SNP detected. SNPs were found by comparing mapping results of the strains to the *L*. *infantum* JPCM5 reference genome. Bootstrapping (n = 100) was used.

WGS resulted in cleaned paired-end reads of 17–38*10^6^ reads per sample, high assembly rate of 98.13–98.52% and coverage of x99-x218. Maximum likelihood analysis including all SNP’s found (including 9,969 positions, S3 Table) resulted in the resistant and susceptible clonal strains dividing into two distinct clusters (Fig 3).

## Discussion

*Leishmania infantum* resistance to allopurinol may pose a combined veterinary and public health threat. Drug resistance can result in infected dogs having an uncontrolled high parasite load and being parasitemic for longer periods, increasing both the impact of the disease on the canine host and the potential for transmission via sandflies to humans [9, 10]. Valuable molecular and biochemical information can be obtained by analyzing resistant field isolates. The *in-vitro* generation of drug resistance is a useful complementary tool for elucidating the mechanisms of resistance formation, especially when a genetic basis is suspected and sought [11–15]. As a first step in exploring resistance to allopurinol we constructed an *in-vitro* promastigote model that allowed monitoring the progression of resistance development under drug pressure over time, with less variables and complexity compared to an intracellular amastigote based model. This same approach, when applied in studies of antimonials [16] and miltefosine [17], resulted in the identification of genetic changes found also in resistant amastigote strains [18, 19]. In the present study, we applied drug pressure of up to 10 times higher (900μg/mL) than the initial IC_50_ of the two induced promastigote cultures, a drug level compared to the average IC_50_ level found previously for resistant clinical isolates (996±372μg/mL) [3]. This experimental setup has induced or selected for a considerable increase in allopurinol resistance, resulting in IC_50_ levels of up to 20 folds higher than initial level, comparable to levels measured for allopurinol-resistant parasites isolated from dogs that experienced clinical disease relapse [3]. Three distinct stages were discerned by monitoring growth rates and IC_50_ values during the induction of resistance. Initially, following introduction of drug pressure parasite growth rates decreased. This was due either to adaptation to the culture medium or the additional stress put on by the drug. Although an increase in IC_50_ values accompanied the decreased growth rate, it was found to be non-significant and shared by both control and test cultures. Therefore, this increase may also represent an adaptation to the culture medium and to the purine sources in particular, affecting the uptake or metabolism of the purine analog allopurinol [20–22]. Following this period of adaptation the growth rates of the isolates cultured with drug at least doubled in parallel to a significant increase in their IC_50_. Since the peak IC_50_ values were measured slightly after maximum drug concentration was reached, this IC_50_ increase was most likely due to genetic adaptations, where the drug pressure selected variants that carried advantageous polymorphisms [15, 23, 24]. The existence of an inherent basis for resistance is supported also by the relatively high correlation found between drug susceptibilities of promastigotes and amastigotes of respective strains. The dynamics of resistance formation seen here fits the suggested model for appearance of pathogen drug resistance in infectious diseases following treatment, which includes emergence, establishment, increase and equilibrium of mutations promoting growth and survival [25]. Noteworthy is the significant 2–3 folds decline in IC_50_ values following the peak values seen in both NT4.L and NT5.L. This decline occurred when the drug concentration in the medium was maintained constant at maximal levels, leaving IC_50_ values still significantly higher compared to most time points during the adaptation phase. This phenomenon might reflect the result of intra-clonal mechanisms such as negative sign epistasis between mutations or may be caused by inter-clonal interaction in mixed cultures in response to prolonged exposure to high drug concentrations, such as clonal interference [25, 26]. In support of the latter explanation, the IC_50_ values of clones generated from a culture under drug pressure at individual time points revealed significant heterogeneity, both when promastigotes or amastigotes were tested. As a rule, clonality is well recognized in *Leishmania* and was suggested to play a role in its evolution [15]. Albeit the heterogeneity in IC_50_ values within cultured populations, both cultures (NT4.L, NT5.L) demonstrated very similar patterns during the development of allopurinol resistance, both with respect to IC_50_ values and growth rates. This suggests that the process of adaptation to drug pressure may have been similar in both independent cultures.

In conclusion, this study describes the successful induction of allopurinol resistance in *L*. *infantum*, under drug pressure. The model may facilitate studies on the mechanisms, pathways and genetics of allopurinol resistance in parasite populations, as well as identification and monitoring of resistance in clinical isolates.

## Supporting information

S1 Table IC_50_ values for isolates and respective clones at selected time points during induction of resistance. (PDF)

S2 Table Allopurinol susceptibilities of intracellular amastigotes strains. (PDF)

S3 Table Total single nucleotide polymorphisms (SNP's) found for the 6 clones sent for whole genome sequencing. (XLSX)
