# Supplementary material for: Induction of allopurinol resistance in Leishmania infantum isolated from dogs
Source: PLoS Negl Trop Dis. 2017 Sep 11;11(9):e0005910. doi: 10.1371/journal.pntd.0005910 (PMC5608428; doi:10.1371/journal.pntd.0005910)
Supplement: S1 Table — (PDF) [file pntd.0005910.s001.pdf]

Table S1. IC_{50} values for isolates and respective clones at selected time points during induction of resistance.
IC_{50} values of clones presented a 2-4 fold variation at all time points, with significant differences between clones at most time points (Tukey-HSD test, $p<0.05$).
NT4.L, NT5.L - induced resistant cultures. NT4, NT5 - Allopurinol free control cultures.

Isolate	Parent/clone/ control	Day	Allopurinol IC_{50} (μg/mL)	Average	Tukey-HSD
NT4	control	0*	119	105	BC
NT4	control	0*	91		
NT4.L	Parent	4 (R1)	144	154	BC
NT4.L	Parent	4 (R2)	182		
NT4.L	C1	4	76	87	C
NT4.L	C1	4	98		
NT4.L	C2	4	187	215	B
NT4.L	C2	4	243		
NT4.L	C3	4	355	380	A
NT4.L	C3	4	405		
NT4.L	C4	4	141	117	BC
NT4.L	C4	4	93		
NT4.L	C5	4	167	151	BC
NT4.L	C5	4	135		
NT4	control	28	382	361	A
NT4	control	28	339		
NT4.L	parent	28	329	286	A
NT4.L	parent	28	371		
NT4.L	C1	28	85	108	B
NT4.L	C1	28	131		
NT4.L	C2	28	152	136	B
NT4.L	C2	28	120		
NT4.L	C3	28	281	270	A
NT4.L	C3	28	259		
NT4.L	C4	28	370	380	A
NT4.L	C4	28	390		
NT4.L	C5	28	324	293	A
NT4.L	C5	28	262		
NT4	control	94*	341	322	E
NT4	control	94*	303		
NT4.L	Parent	83	978	1240	CDE
NT4.L	Parent	83	1226		
NT4.L	C1	83	2082	1890	ABC

Isolate	Parent/clone/ control	Day	Allopurinol IC_{50} (μg/mL)	Average	Tukey-HSD
NT5	control	0*	101	93	D
NT5	control	0*	85		
NT5.L	Parent	4	220	401	BCD
NT5.L	Parent	4	336		
NT5.L	C1	4	583	502	ABC
NT5.L	C1	4	421		
NT5.L	C2	4	94	152	D
NT5.L	C2	4	210		
NT5.L	C3	4	609	542	AB
NT5.L	C3	4	475		
NT5.L	C4	4	69	87	D
NT5.L	C4	4	105		
NT5.L	C5	4	113	92	D
NT5.L	C5	4	71		
NT5.L	C6	4	242	200	D
NT5.L	C6	4	158		
NT5.L	C7	4	301	248	BCD
NT5.L	C7	4	195		
NT5.L	C8	4	188	214	CD
NT5.L	C8	4	240		
NT5.L	C9	4	356	332	ABCD
NT5.L	C9	4	308		
NT5.L	C10	4	704	610	A
NT5.L	C10	4	516		
NT5	control	28	382	405	A
NT5	control	28	427		
NT5.L	parent	28	478	312	A
NT5.L	parent	28	318		
NT5.L	C1	28	562	495	A
NT5.L	C1	28	428		
NT5.L	C2	28	366	308	A
NT5.L	C2	28	250		
NT5.L	C3	28	440	414	A

NT4.L	C1	83	1698		
NT4.L	C2	83	481	508	DE
NT4.L	C2	83	535		
NT4.L	C3	83	2437	2220	A
NT4.L	C3	83	2003		
NT4.L	C4	83	1936	1746	ABC
NT4.L	C4	83	1556		
NT4.L	C5	83	492	506	DE
NT4.L	C5	83	520		
NT4.L	C6	83	2031	1931	AB
NT4.L	C6	83	1831		
NT4.L	C7	83	1612	1461	ABC
NT4.L	C7	83	1310		
NT4.L	C8	83	1010	1154	BCD
NT4.L	C8	83	1298		
NT4.L	C9	83	1058	1240	BCD
NT4.L	C9	83	1422		
NT4.L	C10	83	1392	1223	BCD
NT4.L	C10	83	1054		
NT4	control	104	237	251	C
NT4	control	104	264		
NT4.L	parent	104	1922	1325	A
NT4.L	parent	104	1540		
NT4.L	C1	104	1882	1668	AB
NT4.L	C1	104	1454		
NT4.L	C2	104	701	580	C
NT4.L	C2	104	459		
NT4.L	C3	104	1284	1123	ABC
NT4.L	C3	104	962		
NT4.L	C4	104	944	829	BC
NT4.L	C4	104	714		
NT4.L	C5	104	721	904	ABC
NT4.L	C5	104	1087		
NT4	control	142	181	170	D
NT4	control	142	158		
NT4.L	parent	142	1026	847	BC
NT4.L	parent	142	798		
NT4.L	C1	142	372	457	CD
NT4.L	C1	142	542		

NT5.L	C3	28	388		
NT5.L	C4	28	438	378	A
NT5.L	C4	28	318		
NT5.L	C5	28	621	546	A
NT5.L	C5	28	471		
NT5	control	48	192	224	D
NT5	control	48	255		
NT5.L	parent	48	1063	1204	BC
NT5.L	parent	48	781		
NT5.L	C1	48	1495	1398	B
NT5.L	C1	48	1301		
NT5.L	C2	48	561	518	CD
NT5.L	C2	48	475		
NT5.L	C3	48	1115	997	BC
NT5.L	C3	48	879		
NT5.L	C4	48	261	320	D
NT5.L	C4	48	379		
NT5.L	C5	48	2113	2003	A
NT5.L	C5	48	1893		
NT5	control	86	294	333	D
NT5	control	86	371		
NT5.L	parent	86	1912	1694	B
NT5.L	parent	86	1742		
NT5.L	C1	86	1224	1113	C
NT5.L	C1	86	1002		
NT5.L	C2	86	1971	1854	B
NT5.L	C2	86	1737		
NT5.L	C3	86	2999	2822	A
NT5.L	C3	86	2645		
NT5.L	C4	86	1817	1921	B
NT5.L	C4	86	2025		
NT5.L	C5	86	1462	1648	BC
NT5.L	C5	86	1834		
NT5	control	111	150	174	F
NT5	control	111	198		
NT5.L	parent	111	804	715	AB
NT5.L	parent	111	756		
NT5.L	C1	111	551	538	CDE
NT5.L	C1	111	525		

NT4.L	C2	142	612	556	CD
NT4.L	C2	142	500		
NT4.L	C3	142	488	547	CD
NT4.L	C3	142	606		
NT4.L	C4	142	831	988	BC
NT4.L	C4	142	1145		
NT4.L	C5	142	1138	985	BC
NT4.L	C5	142	832		
NT4.L	C6	142	713	812	BC
NT4.L	C6	142	911		
NT4.L	C7	142	1299	1204	B
NT4.L	C7	142	1109		
NT4.L	C8	142	2081	1893	A
NT4.L	C8	142	1705		
NT4.L	C9	142	783	898	BC
NT4.L	C9	142	1013		
NT4.L	C10	142	376	411	CD
NT4.L	C10	142	446		

NT5.L	C2	111	813	752	ABC
NT5.L	C2	111	691		
NT5.L	C3	111	901	869	A
NT5.L	C3	111	837		
NT5.L	C4	111	589	650	ABCDE
NT5.L	C4	111	711		
NT5.L	C5	111	685	713	ABCD
NT5.L	C5	111	741		
NT5.L	C6	111	561	531	CDE
NT5.L	C6	111	501		
NT5.L	C7	111	441	472	E
NT5.L	C7	111	503		
NT5.L	C8	111	799	752	ABC
NT5.L	C8	111	705		
NT5.L	C9	111	476	519	DE
NT5.L	C9	111	562		
NT5.L	C10	111	687	638	BCDE
NT5.L	C10	111	589		

* closest available control data point
