# Supplementary material for: Induction of allopurinol resistance in Leishmania infantum isolated from dogs
Source: PLoS Negl Trop Dis. 2017 Sep 11;11(9):e0005910. doi: 10.1371/journal.pntd.0005910 (PMC5608428; doi:10.1371/journal.pntd.0005910)
Supplement: S2 Table — (PDF) [file pntd.0005910.s002.pdf]

Table S2. Allopurinol susceptibilities of intracellular amastigote strains. Outcomes are presented as percent inhibition in cultures treated with 300µg/mL allopurinol relative to untreated controls. An induced resistant culture, five of its respective clones and its drug-free control culture were tested for each parental line (NT4.L and NT5.L) at one time point (day 104 and 86, respectively). Values indicated by distinct letters differ significantly (Tukey HSD test, $p<0.05$). R1,R2 – replicates.

	Average amastigote count per 100 macrophages								Percent inhibition$^{\$}$			
	treated				untreated							
	R1	R2	mean	stdev	R1	R2	mean	stdev	R1	R2	mean	stdev
**NT4.L day 104**												
**NT4 (control)**	51	62	56.5	7.8	75	81	78.0	4.2	32	23	27.7^{A}	6.0
**NT4.L**	105	95	100.0	7.1	111	97	105.0	9.9	5	2	3.7^{B}	2.4
**clone 1**	62	80	71.0	12.7	62	85	73.5	16.3	0	6	2.9^{B}	4.2
**clone 2**	68	60	64.0	5.7	87	75	81.0	8.5	22	20	20.9^{AB}	1.3
**clone 3**	88	102	95.0	9.9	91	109	100.0	12.7	3	6	4.9^{B}	2.2
**clone 4**	89	80	84.5	6.4	105	85	95.0	14.1	15	6	10.6^{B}	6.6
**clone 5**	77	93	85.0	11.3	85	101	93.0	11.3	9	8	8.7^{B}	1.1
**NT5.L day86**												
**NT5 (control)**	105	90	97.5	10.6	132	117	124.5	10.6	20	23	21.8^{A}	1.9
**NT5.L**	109	135	122.0	18.4	121	152	136.5	21.9	10	11	10.6^{AB}	0.9
**clone 1**	105	119	112.0	9.9	113	129	121.0	11.3	7	8	7.4^{B}	0.5
**clone 2**	119	89	104.0	21.2	125	91	108.0	24.0	5	2	3.5^{B}	1.8
**clone 3**	119	135	127.0	11.3	122	132	127.0	7.1	2	-2	0.1^{B}	3.3
**clone 4**	138	110	124.0	19.8	155	112	133.5	30.4	11	2	6.4^{B}	6.5
**clone 5**	118	151	134.5	23.3	120	151	135.5	21.9	2	0	0.8^{B}	1.2

$^{\$}$ Percent Inhibition = [1- (Number of amastigotes in allopurinol treated DH-82 cells / Number of amastigotes in control untreated DH-82 cells)] x 100.
